# Reconstruction of the Lateral Collateral Ligament Using a Suture Tape Anchor for Iatrogenic Hallux Varus

**DOI:** 10.1155/2021/8784421

**Published:** 2021-10-26

**Authors:** Akinori Nekomoto, Tomoyuki Nakasa, Yasunari Ikuta, Junichi Sumii, Nobuo Adachi

**Affiliations:** ^1^Department of Orthopaedic Surgery, Graduate School of Biomedical and Health Sciences, Hiroshima University, Japan; ^2^Medical Center for Translational and Clinical Research, Hiroshima University Hospital, Japan; ^3^Sports Medical Center, Hiroshima University Hospital, Japan

## Abstract

Iatrogenic hallux varus is a difficult complication of hallux valgus surgery. Although tendon transfer combined with bony correction is performed for hallux varus, tendon transfer has several disadvantages, such as the complicated nature of the procedure and the donor site morbidity. We describe the case of a 70-year-old woman with iatrogenic hallux varus treated by lateral collateral ligament (LCL) reconstruction using a suture tape anchor with bony correction. Tarsometatarsal joint arthrodesis was performed to correct the narrow intermetatarsal angle (IMA), and the varus deformity of the great toe at the metatarsophalangeal joint was corrected by anatomical reconstruction of the LCL using the suture tape anchor. One year postoperatively, the Japanese Society for Surgery of the Foot Hallux Metatarsophalangeal-Interphalangeal Scale had improved from 37 to 90 points. Radiography confirmed that the hallux valgus angle had been corrected from -24° to 4° and the IMA from 0° to 8°. Reconstruction of the LCL using suture tape anchor is an easy procedure for iatrogenic hallux varus which can achieve good stabilization.

## 1. Introduction

Although the reported incidence of iatrogenic hallux varus is 2% to 15.4% after hallux valgus surgery, this complication remains challenging to treat because of several surgical factors including loss of osseous support, overcorrection of the proximal articular set angle or intermetatarsal angle (IMA), and muscular imbalance of the proximal phalangeal base [1–5]. Arthrodesis of the first metatarsophalangeal (MTP) joint for iatrogenic hallux varus has historically provided analgesia with no recurrence of severe instability [1]. However, motion at the first MTP joint is essential for activities requiring push-off power such as running and jumping. Therefore, other options have recently been introduced, including reverse osteotomies and tendon transfer procedures [1]. However, tendon transfer has several problems such as high demand techniques to adjust tendon and uncertain long-term clinical outcomes due to the loss of the muscle tension [1]. Osteotomies to correct IMA should be considered the healthy osteotomy site to avoid the nonunion in the case which the prior osteotomy was performed [1]. Therefore, an easier and more convenient technique to preserve the joint function of the first MTP is needed. Joint preservation surgery in the first MTP joint should be performed unless there is a severe osteoarthritis (OA) change in the joint, especially in iatrogenic case [6]. The two most important outcomes in joint preservation of the first MTP joint after hallux valgus surgery are a balanced MTP joint and a corrected IMA. The IMA is corrected by osteotomy but reobtaining balancing in the first MTP is challenging. Recently, suture tape devices have been developed and widely used to stabilize unstable joints [7–9]. We describe the case of a patient whose iatrogenic hallux varus was treated with tarsometatarsal (TMT) joint arthrodesis and lateral collateral ligament (LCL) reconstruction of the first MTP joint using a suture tape anchor device, with satisfactory clinical outcomes.

## 2. Case

A 70-year-old woman underwent a modified Mann's procedure for her right hallux valgus at a previous hospital three years ago. Postoperatively, the hallux varus deformity occurred with dorsal dislocation of the second phalanx at the MTP joint. She complained of severe pain at the metatarsal head of the plantar side of the second toe. Therefore, she was referred to our hospital for further treatment.

**Figure 1:**
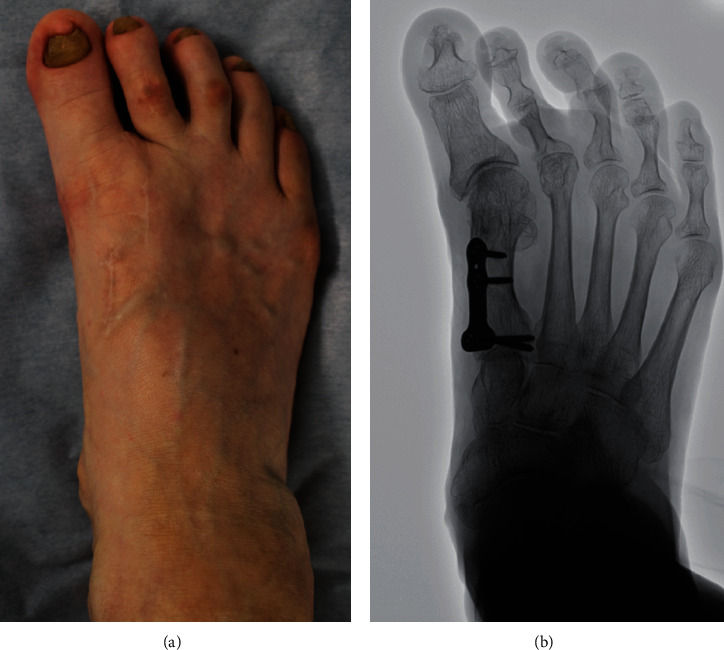
Preoperative appearance (a) and plain radiograph in the standing position (b). The hallux valgus angle was -24° and the intermetatarsal angle 0°.

Her right foot exhibited a hallux varus deformity, and the second and third toes were dislocated dorsally (Figure 1(a)). Skin erosion at the dorsal aspect of the proximal interphalangeal joint of the second and third toes was observed. The range of motion (ROM) of the first MTP joint was 40° in dorsiflexion and 10° in plantarflexion. There was a tender callosity at the plantar aspect of the second metatarsal head. On plain radiographs, the hallux valgus angle was -24°, and the IMA was 0° (Figure 1(b)). The joint space at the first MTP joint was maintained.

**Figure 2:**
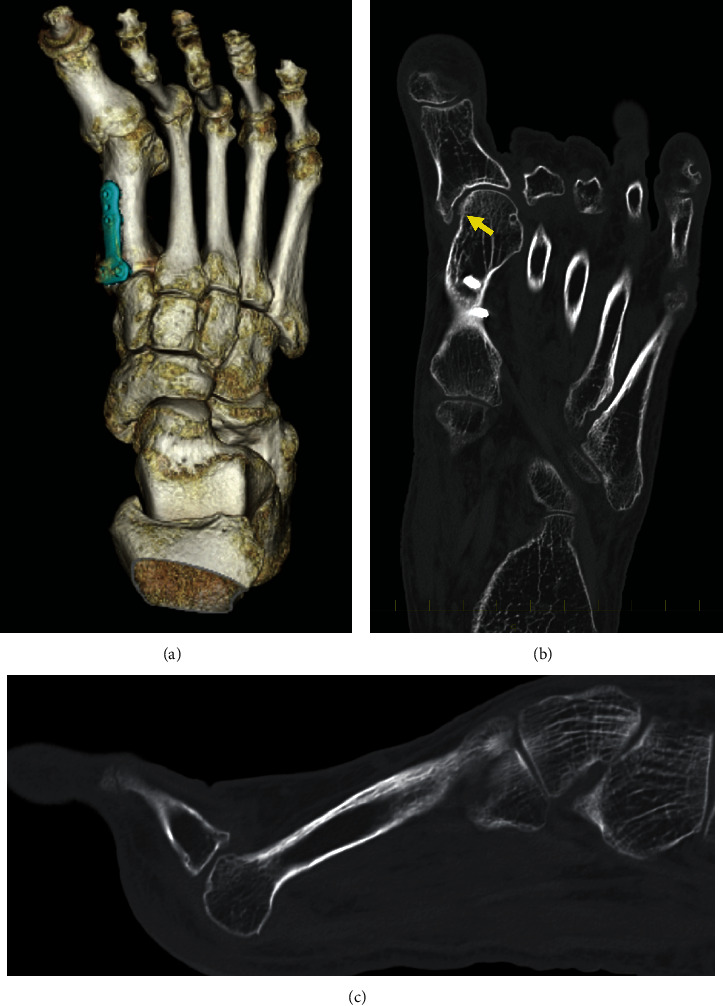
Preoperative CT images. (a) 3DCT image. (b) Coronal image. Arrow indicates decreasing the surface area of the medial side of the articular surface at the first metatarsal head by resection of the bony prominence. (c) Dorsal dislocation of the 2nd toe. CT: computed tomography; 3DCT: three-dimensional computed tomography.

Three-dimensional computed tomography (3DCT) revealed the hallux varus deformity and dorsal dislocation of the second toe (Figure 2(a)). Osteophyte formation was observed at the proximal end of the phalanx. On the coronal image, the surface area of the medial side of the articular surface at the first metatarsal head was decreased by the resection of the bony prominence (Figure 2(b)). Because her symptoms such as pain and gait disturbance had worsened, surgery was performed.

**Figure 3:**
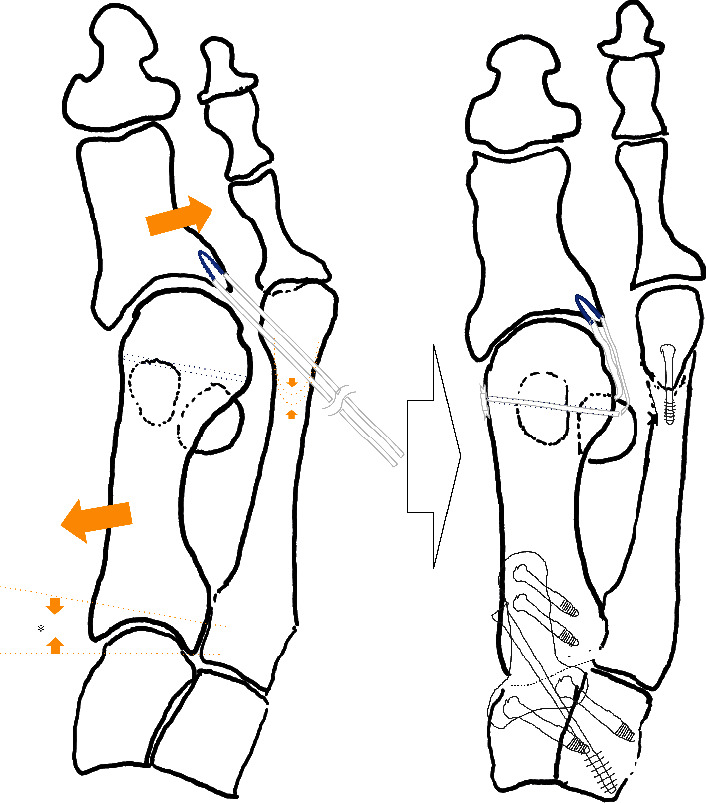
Schema of the surgical procedure.

The patient was placed in a supine position under saphenous and sciatic nerve block, and a pneumotourniquet was applied to a lower leg. To correct the hallux varus deformity after the proximal osteotomy, we planned three steps, namely, correction of the IMA by corrective arthrodesis of the TMT joint, a shortening osteotomy of the second metatarsal bone, and reconstruction of the LCL of the first MTP joint (Figure 3).

First, the fixation plate and screws inserted at the proximal side of the metatarsal bone during the modified Mann procedure were removed using the previous skin incision. The skin incision was extended 1.5 cm proximally, and the first TMT joint was exposed to facilitate arthrodesis. The metatarsal base was resected in a wedge shape, with the medial side 4 mm from the TMT joint. The medial cuneiform bone was cut at a thickness of approximately 1.5 mm parallel to the line of the articular surface to prepare for the arthrodesis to correct the IMA and pronation deformity. The TMT joint was tentatively fixed with a 1.5 mm Kirschner wire, and the appropriate IMA aiming at 8° was confirmed under fluoroscopy. Then, the TMT joint was fixed using a cannulated cancellous screw (Asnis microscrew 3.0 mm, Stryker, Mahwah, NJ, USA) to apply a compression force, and additional fixation was achieved with a locking plate with screws (Variax Foot, Oblique T plate, Stryker, Mahwah, NJ, USA).

Second, a 3 cm incision was made on the medial side of the first MTP joint and a 3 cm incision at the first dorsal intermetatarsal area using the previous skin incision. To correct of the second toe at the MTP joint, a shortening osteotomy of the second metatarsal bone at the distal side was performed. The metatarsal bone was resected obliquely in a sagittal plane to a length of 3 mm and fixed with a cannulated cancellous screw (Asnis microscrew 2.0 mm, Stryker, Mahwah, NJ, USA). Then, reduction of the second toe was performed, and the MTP joint was temporarily fixed with a 1.2 mm Kirschner wire (*K*-wire).

**Figure 4:**
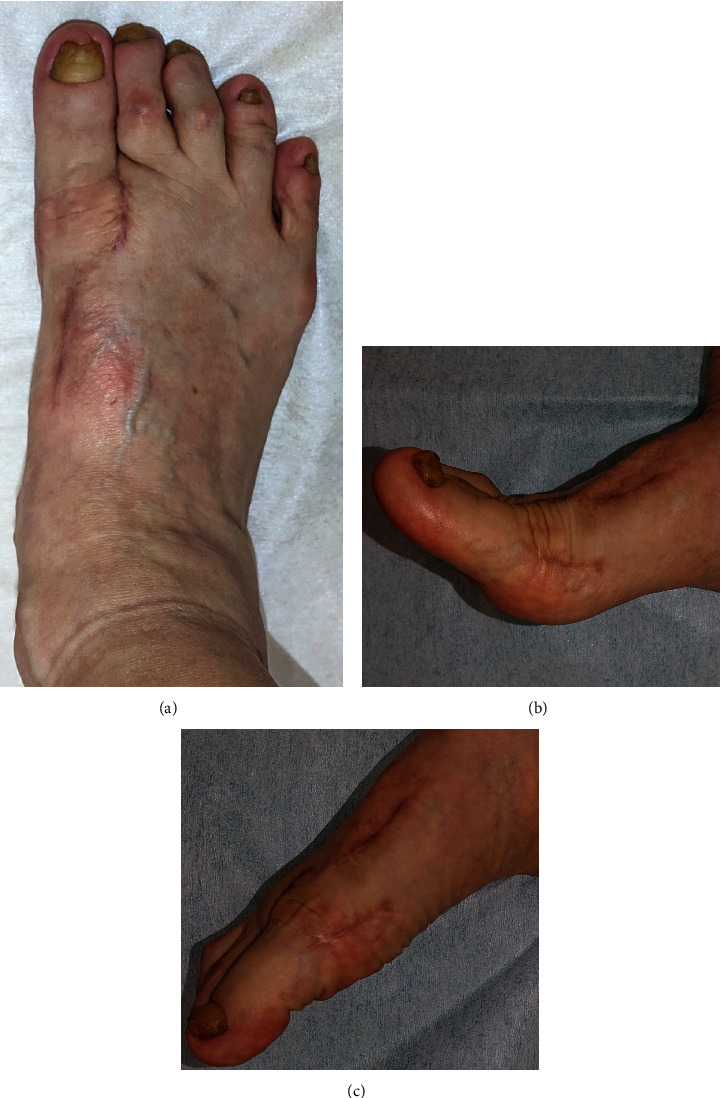
Appearance of the foot (a) and active motion of the great toe at one year postoperatively. (b) Dorsiflexion. (c) Plantarflexion.

**Figure 5:**
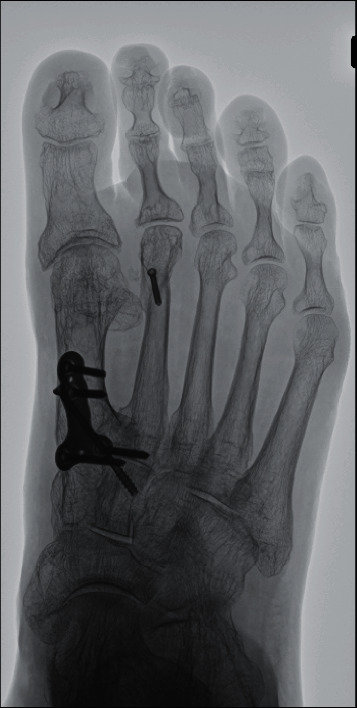
Radiograph one year postoperatively. The hallux valgus angle was 4° and the intermetatarsal angle 8°.

Finally, the LCL reconstruction in the first MTP joint was performed using suture tape anchor (DX Suture Anchor with 1.3 mm Fiberwire Suture Tape, Arthrex, Inc., Naples, FL.). The adhesion of the abductor hallucis muscle was released through the lateral incision, and the joint capsule on the lateral side was dissected in the U shape including the loosened LCL. The footprint of the LCL at the proximal phalanx was exposed, and an anchor with the suture tape was inserted. Then, two bone tunnels were created at the attachment of the proximal end of the LCL in the metatarsal head using a 1.2 mm *K*-wire, and the suture tape was pulled out from lateral to medial by suture relay. With the holding of the great toe at the appropriate alignment aiming at 5° of the hallux valgus angle, poly-L-lactic acid (PLLA) pins (SuperFixsorb, Depuy, Rayhnam, MA) were inserted into the bone tunnels to fix the suture tape while it was manually pulled to the maximum tension. Prior to the fixation of the suture tape, the first MTP joint motion was confirmed to determine the degree of the tension. Then, suture tapes were sutured to the medial capsule. Keeping the good alignment of the great toe, the first MTP joint was temporarily fixed with a 1.2 mm *K*-wire. The U-shape lateral capsule was sutured with 2-0 thread at the lateral side of the metatarsal head while maintaining tightness. Postoperatively, a short leg cast was applied for three weeks. Full-weight bearing was permitted from two weeks using a short leg cast with a rubber walking heel. Three weeks postoperatively, the cast was removed, and the patient was administered an insole and allowed to walk without a propulsive toe off. Eight weeks postoperatively, the patient was allowed to be active without any particular limitation. One year postoperatively, there were no residual complaints of pain. The hallux valgus deformity had improved to the extent that she could wear commercial shoes without difficulty (Figure 4(a)). The ROM of the first MTP joint was 65° in dorsiflexion and 15° in plantarflexion (Figures 4(b) and 4(c)). The Japanese Society for Surgery of the Foot (JSSF) Hallux Metatarsophalangeal-Interphalangeal Scale had improved from 37 to 90 points [10, 11]. On the patient-reported SAFE-Q score, all subscale scores improved from pre- to postoperatively, as follows: pain and pain-related questions, from 18.9 to 71.7 points; physical functioning and daily living, from 34.1 to 59.1 points; social functioning, from 54.2 to 75 points; shoe-related questions, from 0 to 58.3 points; general health and well-being, from 25 to 60 points; and sports, from 0 to 1.1 points [12, 13]. Radiographic findings showed that the hallux valgus angle had corrected from -24° to 4°, and the IMA had corrected from 0° to 8° (Figure 5). Progression of OA of the first MTP joint was not observed.

## 3. Discussion

Several studies related to the difficulty of the surgical treatment of the iatrogenic hallux varus have been published. However, we successfully treated it using a suture tape anchor with an easy technique. One year postoperatively, improvement of ROM of the first MTP joint was obtained with good alignment, suggesting that an LCL reconstruction using suture tape could achieve physiological and anatomical repair of the MTP joint. The tension of the LCL appears appropriate because progression of OA of the first MTP joint was not observed, and there was good ROM of the great toe.

Various surgical treatments have been performed for iatrogenic hallux varus. In cases of OA with pain and stiffness in the MTP joint, arthrodesis is recognized as the most appropriate treatment [14]. Indeed, MTP arthrodesis is reliable because there is no risk of recurrence or improvement of the pain at the MTP joint. However, in cases without OA change and with maintenance of the ROM, joint-preserving surgery should be performed. The first-line procedure is medial capsule release in the retracted part of the first MTP joint including the abductor hallucis tendon, which provides the deforming force toward hallux varus [15]. Subsequently, fibrosis is released in the first intermetatarsal space to restore the intermetatarsal divergence and valgus phalanx positioning. These procedures are convenient, but insufficient to maintain reduction, and usually require additional techniques such as tendon transfer and bony correction [16]. Tendon transfer with the abductor hallucis or extensor hallucis longus tendon aims to compensate for the LCL and uses either a dynamic technique with the muscle body or a static technique without. Although this may achieve stabilization of the lateral side of the MTP, there are several disadvantages. In dynamic transfer, tension is difficult to adjust, and there is a risk of long-term tension loss, while adjusting the tendon in static transfer is simplified and stabilizes over time. However, incorrect positioning of the transfer in either method can make the joint nonfunctional, and the procedures are complicated. Above all, donor site morbidity, including functional loss and surgical invasion, should be considered. To avoid these complications, we performed the LCL reconstruction using suture tape.

Recently, joint stabilization techniques using artificial ligaments have been developed to achieve the reconstruction of the collateral ligament in various joint [7–9]. Cho et al. demonstrated the stabilization of the first MTP joint in a patient with chronic varus instability using suture tape [17]. In their procedure, 2.7 mm bone tunnels were created at the proximal phalanx and the metatarsal bone under fluoroscopy, whereafter suture tape was fixed using the 3.0 mm biotenodesis screws with congruent reduction. Other reports have shown the utility of a suture button device (mini TightRope^R^, Arthrex) for medial instability of the first MTP joint and traumatic hallux varus deformity [18–20]. Using these devices will enable less invasive joint stabilization surgery with a lower recurrence rate. We believe that adding these devices to the treatment of iatrogenic hallux varus deformity will improve the clinical outcome even if the pathology is complicated. In addition, the anchor system with suture tape allowed us to insert the suture tape precisely at the LCL attachment of the proximal phalanx, which is expected to facilitate more anatomical motion of the great toe.

In addition to the LCL reconstruction using suture tape, TMT joint fusion to correct the IMA was performed because a closed IMA is one of the factors for iatrogenic hallux varus [1]. Generally, IMA < 6° after correction of the hallux valgus should be revised to reopen the IMA. We aimed at an IMA of over 6°, but excessive correction of the IMA leads to the recurrence of hallux valgus. Therefore, we aimed at an IMA of 8° by adjusting the osteotomy at the TMT joint. This angle is a physiological IMA; however, it risks recurrence of the hallux varus due to the slight bony defect at the medial side of the metatarsal head. LCL reconstruction using suture tape provided a strong constraint in the varus direction, and we obtained good clinical outcomes without recurrence. We believe that ligament reconstruction using suture tape could be a useful procedure for chronic varus instability of the first MTP joint.

## 4. Conclusion

In conclusion, we described the successful treatment of an iatrogenic hallux varus using LCL reconstruction with suture tape anchor and TMT joint fusion. Reconstruction of the LCL with a suture tape anchor is an effective technique for iatrogenic hallux varus with little progression of arthritis, and this technique can be considered for the treatment of iatrogenic hallux varus.

## Data Availability

Data supporting the results of the manuscript are included within the manuscript.
